# RFMirTarget: Predicting Human MicroRNA Target Genes with a Random Forest Classifier

**DOI:** 10.1371/journal.pone.0070153

**Published:** 2013-07-26

**Authors:** Mariana R. Mendoza, Guilherme C. da Fonseca, Guilherme Loss-Morais, Ronnie Alves, Rogerio Margis, Ana L. C. Bazzan

**Affiliations:** 1 Instituto de Informática, Universidade Federal do Rio Grande do Sul, Porto Alegre, Rio Grande do Sul, Brazil; 2 Centro de Biotecnologia, Universidade Federal do Rio Grande do Sul, Porto Alegre, Rio Grande do Sul, Brazil; 3 Instituto Tecnológico Vale Desenvolvimento Sustentável, Belém, Pará, Brazil; The University of Queensland, Australia

## Abstract

MicroRNAs are key regulators of eukaryotic gene expression whose fundamental role has already been identified in many cell pathways. The correct identification of miRNAs targets is still a major challenge in bioinformatics and has motivated the development of several computational methods to overcome inherent limitations of experimental analysis. Indeed, the best results reported so far in terms of specificity and sensitivity are associated to machine learning-based methods for microRNA-target prediction. Following this trend, in the current paper we discuss and explore a microRNA-target prediction method based on a random forest classifier, namely RFMirTarget. Despite its well-known robustness regarding general classifying tasks, to the best of our knowledge, random forest have not been deeply explored for the specific context of predicting microRNAs targets. Our framework first analyzes alignments between candidate microRNA-target pairs and extracts a set of structural, thermodynamics, alignment, seed and position-based features, upon which classification is performed. Experiments have shown that RFMirTarget outperforms several well-known classifiers with statistical significance, and that its performance is not impaired by the class imbalance problem or features correlation. Moreover, comparing it against other algorithms for microRNA target prediction using independent test data sets from TarBase and starBase, we observe a very promising performance, with higher sensitivity in relation to other methods. Finally, tests performed with RFMirTarget show the benefits of feature selection even for a classifier with embedded feature importance analysis, and the consistency between relevant features identified and important biological properties for effective microRNA-target gene alignment.

## Introduction

MicroRNAs (miRNAs) are non-coding RNAs of approximately 22 nucleotides (nt) in length that act as an important post-transcriptional mechanism of gene expression regulation via translational repression or degradation of target mRNAs [1], [2]. In both animals and plants, miRNAs are formed after a longer primary transcript (pri-miRNA) by two sequential cleavages, mediated, respectively, by a nuclear and a cytoplasmic RNase III. These processing steps yield a 60
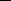
70 nt stem-loop miRNA precursor (pre-miRNA) and next, after the latter is exported to the cytoplasm, a structure of two single RNA strands that corresponds to the mature miRNA, namely the miRNA:miRNA* duplex.

Due to miRNAs participation in important metabolic processes, such as developmental timing, growth, apoptosis, cell proliferation, defense against viruses [3]–[5], and more recently in tumorigenesis, either as tumor suppressors or oncogenes [6], great efforts have been devoted for the identification of novel miRNAs and targets. Despite the advances in deep sequencing approaches, the use of computational tools is still important for analysis and interpretation of data, among which machine learning (ML) algorithms have been prominent. This approach consists in using known positive and negative examples of miRNA-mRNA associations to train a classifier to distinguish, for instance, real pre-miRNAs from pseudo pre-miRNAs, based on a set of descriptive features extracted from the examples. Among the most commonly applied ML algorithms, one may highlight the use of support vector machine (SVM) [7], [8], random forest [9] and naïve Bayes [10] classifiers.

**Figure 1:**
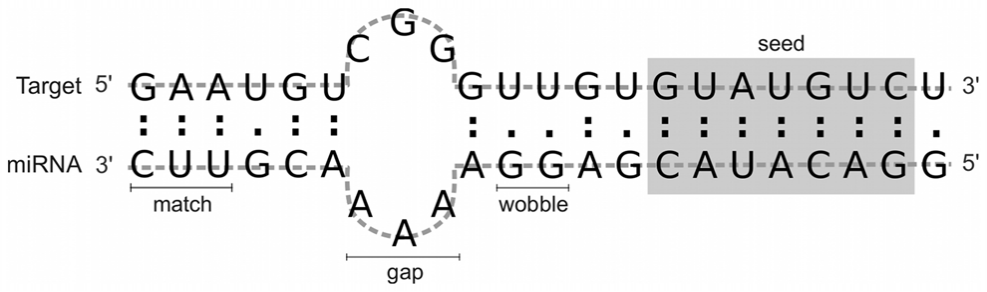
Example of miRNA-target alignment. This schematic representation shows some structural features used for target prediction by the RFMirTarget tool. The seed region, comprising six to eight nucleotides in the 5′ end, is shown in grey. Nucleotides matches are shown by colons, whereas G:U wobble pairs are represented by dots. An example of an alignment gap is also given.

Following this direction, ML-based methods can help in the prediction of miRNA target genes, generating hypotheses regarding miRNA function and potential miRNA:target interactions. However, this is considered to be a more difficult problem, mostly because i) it is hard to distinguish true miRNA-mRNAs hybrids given the millions of possible miRNA-gene combinations and ii) there is still very limited knowledge about the basic mechanisms of microRNA target recognition [11]. Primarily, the interaction of a miRNA and its target occurs by complementarity of their nucleotide sequences, as shown in Fig. 1. Nonetheless, while in plants miRNAs bind their targets with (near) perfect complementarity and mostly in their open read frames [12], in animals, miRNAs sequences have a partial complementarity to their targets and the hybridization may occur in either 3′ untranslated region (3′ UTRs, predominantly) or 5′UTR [13]. Furthermore animals miRNAs contain a region named seed, comprising six to eight nucleotides in the 5′ end, that plays an important role in the correct interaction between the miRNA and its target, showing (almost) strict pairing with the mRNA (Fig. 1). In some cases, however, the 3′ out-seed segment of the miRNA-mRNA alignment can compensate imperfect base pairing in the seed region [14].

The wide variation in animals miRNAs-target standard hybridization has turned this problem into a challenge in the field and motivated the development of several computational methods. The first efforts towards this problem were concentrated in performing predictions based on sequence complementarity and/or favourable miRNA-target duplex thermodynamics [15], [16]. Among the most disseminated tools, miRanda [17], TargetScan [18] and PicTar [19] are complementarity-based methods that first identify potential binding sites by scoring the aligned sequences and analyzing their seed region (in the case of animals), and then evaluate their thermodynamics using, for instance, the Vienna RNA folding package [20]. However, such tools are prone to produce many false positive interactions and are usually better suitable for plants miRNAs, which differently from animals miRNAs, show near to perfect complementarity when binding to their targets. In addition, these tools lack a statistical background model to evaluate the significance of each detected hit [15].

Despite the relative success of the aforementioned tools, ML-based methods, like TargetSpy [11], NBmiRTar [21], miTarget [22], TargetMiner [23], and MultiMiTar [24], have had the best results so far in terms of specificity and sensitivity in the prediction of miRNAs target genes [24]. NBMirTar implements a naïve Bayes classifier and TargetSpy relies in a learning scheme based on boosting, while the remainder are based on the popular framework SVM. Regardless the classifier adopted, ML methods usually analyze descriptive features derived from the interaction between a miRNA and its potential targets and attempt to extract rules of target site recognition, building a classifier upon this information. Common features categories are seed complementarity, thermodynamics stability, presence of multiple target sites and evolutionary conservation among species [1], [25]. Nonetheless, ML-based tools face an ubiquitous problem: in general, the number of known negative examples is much smaller than known positive examples, which impairs the accuracy of classifiers sensitive to class imbalance, such as SVM.

In this paper we discuss and explore the predictive power of RFMirTarget, a ML approach for predicting human miRNAs target genes based on the random forests algorithm. In what concerns the identification of novel miRNAs, for instance, random forest have been successfully applied, outperforming competing algorithms [9]. This efficiency comes from the manner the algorithm profits from ensemble predictions: during training, several trees are grown such that when unlabelled examples are presented to the classifier, each tree votes for the class of new instances and a majority voting is performed to define the predicted class. In spite of its outstanding performance in other classification tasks, to the best of our knowledge random forests have been barely explored as for miRNA target prediction.

Xiao and colleagues [26], for instance, have focused in a systematic analysis of features importance carried with a random forest model, whereas here our goal is to deeply explore the predictive power of the random forests algorithm and perform a comprehensive comparison with other popular classifiers in the field. In our previous work [27], we presented RFMirTarget and discussed its features and training data, as well as preliminary results of our model performance for the prediction of Human miRNAs targets. In this paper, we extend this study in several directions: i) we perform a feature relevance analysis and investigate the effects of feature selection over the predictive accuracy of our model, observing improvement in the overall performance of our classifier, ii) we investigate the impact of feature categories in classification, as well as apply techniques to interpret the ensemble model and the effects of features values over class probabilities, iii) we discuss biological insights provided by the resulting model, highlighting its suitability in identifying biologically relevant features in this classification problem, iv) we test several algorithmic variants such as definition of class weights and distinct permutation methods for the selection of tree’s nodes, showing that the ensemble approach underlying the random forest algorithm seems to reduce its sensitivity to the class imbalance issue, v) we carry a thorough comparison of RFMirTarget against other popular classifiers, proving that the proposed RF model is indeed robust and that its performance is superior than its counterparts methods with statistical significance, vi) we assess the performance of our method in a completely independent test data set of experimentally verified positive and negative examples of miRNA-target and observe a good overall performance and outstanding sensitivity when compared to other miRNA target prediction algorithms.

In what follows we describe our materials and methods, starting by a brief explanation about the random forests algorithm, followed by a description about our features definition and training data set. In the sequence, we discuss our tool’s performance by exploring its predictive power and robustness, as well as comparing it with other classification methods and miRNA-target prediction algorithms.

## Materials and Methods

**Figure 2:**
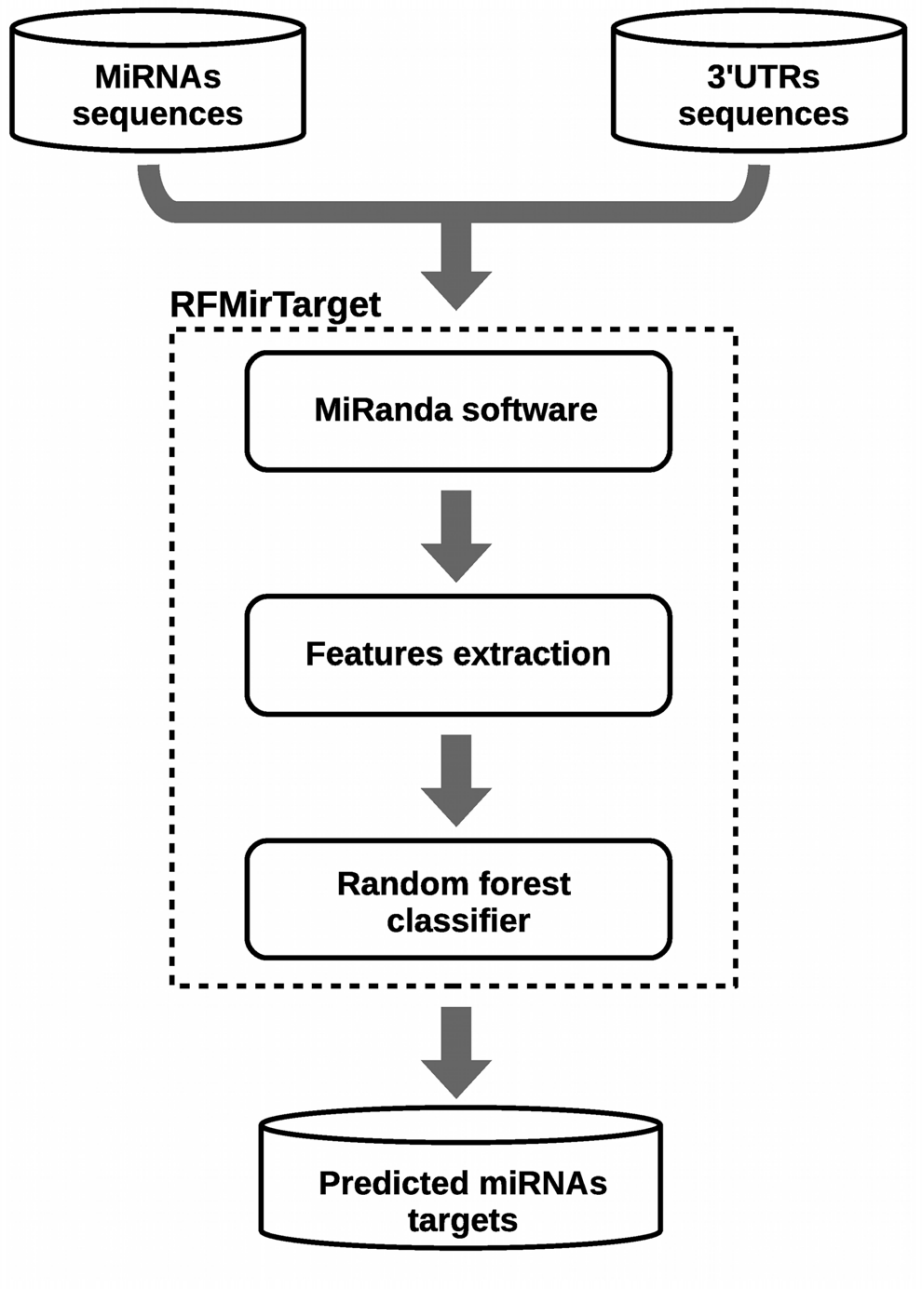
RFMirTarget framework. RFMirTarget is trained upon a set of biologically validated positive and negative miRNA-target examples. This data set is analyzed by miRanda, whose output is processed for features extraction. A random forest model is then built upon these features and can be further used to predict the class of unknown miRNA-target instances.

This section describes the methodology used to build a random forest classifier based on a collected set of biologically validated training data. RFMirTarget is trained with a set of positive and negative examples of miRNA-target pairs that is pre-processed by the software miRanda in order to identify the actual interacting sites between each miRNA-mRNA pair and prepare the data set for feature extraction. The alignments provided by miRanda are the source for features extraction, which in turn are used to train the random forest classifier. Thus, a direct application of our tool is to refine the predictions provided by miRanda. In what follows we explain each of the steps involved in the training process, summarized in Fig. 2.

### Random Forest

Random forest (RF) is a well-known ensemble approach for classification tasks proposed by Breiman [28], [29]. Its basis comes from the combination of tree-structured classifiers with the randomness and robustness provided by bagging and random feature selection. Several decision trees are trained with random bootstrap samples from the original data set (∼2/3 of data) and afterwards, results are combined into a single prediction: for classification tasks, by means of voting; for regression tasks, by averaging all trees results. The fact that the predicted class represents the mode of the classes output by individual trees gives robustness to this ensemble classifier in relation to a single tree classifier.

Another important property of RF classifiers refers to random feature selection. Instead of using all features for growing a tree, Breiman proposed to choose from a random subset of features in order to split at each node. Therefore, at each split step, a constant number of features is randomly chosen from the total set of features, and the best split on this random selection of features, e.g., the one with smallest impurity, is used to split the node.

Tests run by Breiman [29] have revealed that random forests always perform better than the bagging approach previously proposed [28] and also better than Adaboost [30]. However, the benefits of random forests go beyond the good performance. The mechanism applied for growing trees allows the estimation of the most important variables for classification and generates an internal unbiased estimate of the generalisation error during the growth process. These estimative are drawn from the data left out of the bootstrap sample used as training set, named out-of-bag (OOB) data, which corresponds to approximately 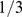 of the instances. Additionally, as random forests are tree-structure classifiers, they inherit some of the interpretability associated to this type of classification model, such as variable relevance estimation, thus making it an appealing choice [9]. The RF model was implemented with the randomForest R package [31].

### Data Set

We train RFMirTarget with experimentally verified examples of human miRNA-target collected by Bandyopadhyay and Mitra [23] for the training process of MultiMiTar [24], a SVM-based miRNA-target prediction system. The data set is composed of 289 biologically validated positive examples extracted from miRecords database [26] and 289 systematically identified tissue-specific negative examples.

To improve the accuracy of the classifier, potential negative examples were detected applying several target prediction algorithms to a set of miRNA-mRNA pairs and selecting those instances predicted as target [23]. As this prediction is based on features drawn from sequence or structural interactions between miRNA and mRNA, it contains many false positives, especially for tissue-specific miRNA. Thus, expression profiling data of a miRNA and its predicted target was used to measure tissue specificity for both of them, and those miRNA-mRNA pairs that are significantly overexpressed in one or a few specific tissue types are chosen as potential negative examples. Next, these potential non-targets are filtered using another independent expression profiling data set and the final set of negative examples is analysed in terms of thermodynamic stability and seed site conservation. We refer the reader to [23] for more details about the data.

### Data Preparation

The data set of positive and negative examples of miRNA-target pairs gathered by Bandyopadhyay and Mitra [23] does not comprises information about the actual site of alignment between miRNAs and their targets. The only information provided is the accession ids for each true and pseudo example of miRNA-target pair. Based on this information, we manually download miRNAs and target sequences from miRBase version 17 (http://www.mirbase.org) and NCBI (http://www.ncbi.nlm.nih.gov) databases, respectively. For miRNAs that can be excised from opposite arms of the same pre-miRNA (–3p or –5p suffix), we download both sequences unless the arm is clearly specified in the data set (for instance, because only the designation hsa-miR-124 is specified by Bandyopadhyay and Mitra, we download both hsa-miR-124-3p and hsa-miR-124-5p miRNAs available at miRBase). We follow the same approach when closely related mature sequences or distinct precursor sequences and genomic loci that express identical mature sequences are available and are not specified in the data set provided by Bandyopadhyay and Mitra (as an example, we download hsa-miR-16-2-3p, hsa-miR-16-5p and hsa-miR-16-1-3p for the instance hsa-miR-16, since no further specification is given by the authors).

Once the miRNAs and target sequences are collected, the binding sites need to be obtained since they are a compulsory information for the features extraction step inherent to ML approaches. As miRNAs are short sequences, they can easily align to multiple sites of their targets. Thus, the use of techniques such as BLAST can result in an extremely large data set, with many biologically unlikely miRNA-mRNA pairs. Indeed, it was already discussed that the performance of BLAST for miRNA target search is controversial [32]. Therefore, to reduce the dimension of our problem and prepare the data set for features extraction, we opt for using the miRanda software [17] to pre-process the data and to obtain the exact miRNA-target binding sites. We apply miRanda in a pairwise fashion, i.e., for every pair of positive and negative examples of miRNA-target genes collected from literature, and post-process its output, extracting a set of descriptive features used to train the model. MiRanda is an algorithm for the detection of potential microRNA target sites in genomic sequences. It runs a score-based algorithm to analyze the complementarity of nucleotides (A:U or G:C) between aligned sequences. First, a dynamic programming local alignment is carried out between the query miRNA sequence and the reference sequence. The scoring matrix allows the occurrence of the non-canonical base-pairing G
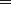
U wobble, which is a non Watson-Crick base pairing with important role in the accurate detection of RNA:RNA duplexes, and is based on the following parameters: +5 for G
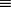
C, +5 for A
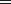
U, +2 for G
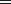
U and -3 for all other nucleotides pairing. The second phase of the algorithm takes alignments that scored above a given threshold and estimates the thermodynamic stability of their RNA duplexes. Finally, detected targets with energy less than an energy threshold are selected as potential targets. Target site alignments satisfying both thresholds (score and energy) are given as miRanda’s output. Therefore, a benefit in employing miRanda to detect binding sites between miRNAs and potential targets is that despite the high probability of finding interaction sites due to some extent to the short length of miRNAs, miRanda filters this information by means of its thresholds. However, we adopt low threshold values such that all reference sequences with the minimal requirements to be considered targets are kept by miRanda, leaving the task of refining results for our tool.

**Figure 3:**
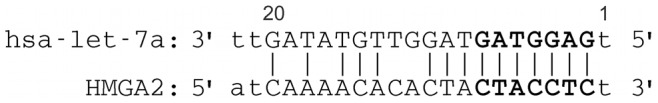
Alignment between hsa-let-7a and its target HGMA2 as predicted by miRanda. The highlighted nucleotides refer to the seed region. In addition, this figure illustrates the nucleotides numbering (1–20) used for position-based features extraction.

Besides the scoring matrix, four empirical rules are applied for the identification of the miRNA binding sites, counting from the first position of the 5′ end of the miRNA: i) no mismatches at positions 2 to 4; ii) fewer than five mismatches between positions 3–12; iii) at least one mismatch between positions 9 and L-5 (where L is the length of the complete alignment); and iv) fewer than two mismatches in the last five positions of the alignment [17]. An example of output provided by miRanda for the miRNA hsa-let-7a and its target HGMA2 is depicted in Fig. 3. To help in the discussion of features definition (next section), we highlight the seed region of the alignment, composed by nucleotides 2 to 8 to count from the 5′ end of the miRNA sequence, as well as we numerate nucleotides 1 and 20, also using as reference the 5′-most position of the miRNA. In this example, we can observe perfect complementarity in the seed region (binding is denoted by the pipe symbol).

After running miRanda on the data set described in the previous section, we obtain 482 positive and 382 negative miRNA-target pairs, which correspond to the training instances used in the building process of our RF classifier. The increase in the number of training instances is due to both the approach followed in data collection and to the possibility of occurrence of multiple binding sites between the same pair of miRNA and candidate target sequence. For instance, the pair hsa-miR-1 and NM_017542.3 indicated in the data set by Bandyopadhyay and Mitra as a positive miRNA-target pair has two possible binding positions according to miRanda analysis (possible binding positions in the reference sequence are 996 to 1017 and 2992 to 3013). At this point we emphasize that albeit our training data set size is different than the one used in [24], they derive from the original data set used for training MultiMitar.

### Features

The negative and positive examples predicted by miRanda consist of the alignment between miRNA-mRNA pairs, as depicted in Fig. 3, based on which the classifier features are extracted. In addition, miRanda provides some alignment properties such as score and length. The set of descriptive features used to train RFMirTarget is divided into five categories: alignment features, thermodynamics features, structural features, seed features and position-based features. In what follows we explain each of the defined features categories.

#### 1. Alignment features

Score and length of the miRNA-target alignment as evaluated by miRanda.

#### 2. Thermodynamics features

Evaluation of the minimum free energy (MFE) of the complete miRNA-target alignment computed by RNAduplex [20].

#### 3. Structural features

Quantification of the absolute frequency of Watson-Crick matches (G:C and A:U pairing) and mismatches (G:U wobble pair, gap and other mismatches) in the complete alignment.

#### 4. Seed features

Evaluation of nucleotides in positions 2–8, to count from the 5′-most position of the miRNA, in terms of thermodynamics (by RNAduplex) and structural alignment properties, i.e., absolute frequency of Watson-Crick matches (G:C and A:U pairing) and mismatches (G:U wobble pair, gap and other mismatches).

#### 5. Position-based features

Evaluation of each base pair from the 5′-most position of the miRNA up to the 20th position of the alignment, assigning nominal values to designate the kind of base pairing in each position: a G:C match, an A:U match, a G:U wobble pair, a gap and a mismatch.

Graphical representations of G:C and A:U matches, G:U wobble pairs and mismatches are given in the example of miRNA-mRNA alignment of Fig. 1. The seed region is also specified. In total, 34 features were drawn from the miRanda output: two alignment features, one thermodynamics feature, five structural features, twenty position-based features and six seed-related features. The complete set of features used by RFMirTarget is summarized in Table 1.

**Table 1:** Summary of features used for classification by RFMirTarget.

	Feature Name	Top 12		Feature Name	Top 12
1	Alignment score	*	18	Position 10	
2	Alignment length		19	Position 11	
3	Minimum free energy of the alignment	*	20	Position 12	
4	G:C’s absolute frequency in the alignment	*	21	Position 13	
5	A:U’s absolute frequency in the alignment	*	22	Position 14	
6	G:U’s absolute frequency in the alignment		23	Position 15	
7	Number of gaps in the alignment		24	Position 16	
8	Number of mismatches in the alignment		25	Position 17	
9	Position 1		26	Position 18	
10	Position 2	*	27	Position 19	
11	Position 3		28	Position 20	
12	Position 4	*	29	Minimum free energy of the seed	*
13	Position 5		30	G:C’s absolute frequency in the seed	*
14	Position 6	*	31	A:U’s absolute frequency in the seed	*
15	Position 7	*	32	G:U’s absolute frequency in the seed	*
16	Position 8		33	Number of gaps in the seed	
17	Position 9		34	Number of mismatches in the seed	

* The top 12 features refer to those features with greatest impact in the predictive accuracy of the RF model, estimated by means of a restricted forward feature selection.

### Performance Assessment

The performance of RFMirTarget is assessed by computing the total prediction accuracy (ACC), specificity (SPE), sensitivity (SEN) and Matthew’s correlation coefficient (MCC) based on the confusion matrix. This matrix quantifies the number of instances in the test set classified as false positive (FP), true positive (TP), false negative (FN) and true negative (TN). In addition, we also plot and evaluate the area under the ROC (Receiver operating characteristic) curve, in which the true positive rate (sensitivity) is plotted in function of the false positive rate (100-specificity) for different decision thresholds. The area under the ROC curve gives us the AUC score, interpreted as the probability that a classifier will rank a randomly chosen positive instance higher than a randomly chosen negative one. Thus, a higher AUC score means a better classification result and a more accurate classifier.
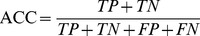
(1)

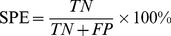
(2)

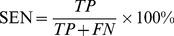
(3)

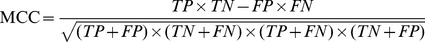
(4)


## Results and Discussion

### Performance of the RF Model

**Figure 4:**
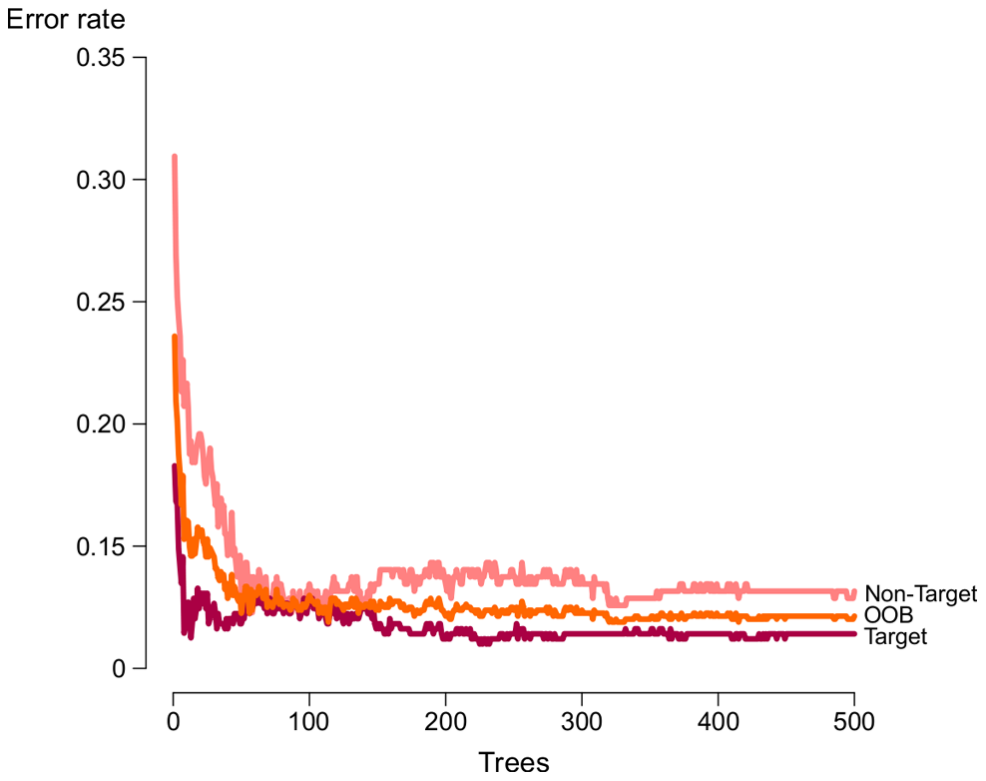
Error rates for RFMirTarget trained with the total set of features. The generalization error decreases as the number of trees in the ensemble prediction increases.

We start the discussion on the results by presenting the performance of a RF classifier trained with the total set of features (Table 1). To train this RF model, as well as further tree-based models presented in this paper, we adopt the standard number of trees suggested by the randomForest R package, namely 500 trees. Previous studies have shown that performance gain is very subtle when doubling or highly increasing the number of trees in the forest, and that the mean and median AUC scores tend to converge asymptotically, thus not justifying the use of very large forests [33]. We experimentally verify this, also observing an stabilisation of error rates around 350 trees (Figure 4). Yet, experiments have shown that there is still a performance gain when adopting 500 trees, thus strengthening our choice regarding the number of trees to be used.

On the other hand, random forests are known to be sensitive to the number of variables (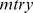) randomly sampled as candidates for splitting at each node during the tree growing process. Thus, we adopt the caret R package [34] to optimize this parameter and perform comparison across models. Resampling is performed to give a better estimative of the error, and based on this estimative we opted for selecting the 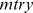 values associated to the simplest model within one standard error of the empirically optimal model, with the purpose of avoiding any overfitting that might be caused by the best performing tuning parameter.

The confusion matrix for the optimized model, averaged over five repetitions of 10-fold cross-validation, is shown in Table 2. Our classifier has an average error rate of 11.8% for the positive class (Target) and 14.1% for the negative class (Non-Target), with standard deviations of 0.60% and 0.79% respectively. The lower efficiency concerning the negative class results in part from the class imbalance problem. In such cases, standard classifiers tend to produce a high predictive accuracy for the majority class and a weaker performance for the minority class. As we will further discuss in this paper, the ensemble approach adopted by RF seems to minimize the difference in classification error between the minority class and the majority class. We evaluate the confusion matrix, obtaining the following performance metrics (with standard deviations in parenthesis): ACC: 87.20 (0.434), SEN: 88.17 (0.604), SPE: 85.84 (0.790 and MCC: 0.737 (0.008).

**Table 2:** Classification performance of RFMirTarget.

		Real	
		Non-Target	Target
**Predicted**	Non-Target	293.6 (2.70)	57 (2.91)
	Target	48.4 (2.70)	425 (2.91)

Confusion matrix for a RF model trained with the total set of 34 features estimated by averaging the results over five repetitions of 10-fold cross-validation. Standard deviations are given in parenthesis.

We compare the results for the 34-features RF model against the performance obtained by RF models trained separately with each of the features categories defined (Table 3). One can observe that, in general, classification based on individual features categories yield very poor classification results as most of them do not have enough generalization power. However, seed and position-based features (categories four and five, respectively) achieve remarkably high and consistent performance in the repeated 10-fold cross-validation process. As previously discussed, the importance of base complementarity in the seed region is a well known factor for miRNA target recognition in Humans. On the other hand, it is also known that additional 3′ pairing increases miRNA functionality and that a single point mutation in the miRNA-mRNA interaction can compromise miRNA’s functioning depending on its position [14], [35]. Thus, position-based features capture the overall quality of the miRNA-target alignment, which in terms of classification perform as well as seed specific positions. In contrast, classification based solely on the minimum free energy of the duplex formation (category two) might include many non-functional target sites [14], justifying the high false positive rate.

**Table 3:** RFMirTarget classification results on different feature subsets.

Feature set	ACC (std)	SPE (std)	SEN (std)	MCC (std)
Cat 1: Alignment (2)	59.34 (0.549)	39.64 (1.066)	73.31 (0.946)	0.136 (0.011)
Cat 2: Thermodynamic (1)	59.39 (1.156)	45.38 (2.211)	69.33 (1.051)	0.150 (0.025)
Cat 3:Structural (5)	67.57 (0.632)	45.38 (0.562)	83.31 (1.074)	0.313 (0.013)
Cat 4: Seed (6)	84.78 (0.407)	82.98 (0.811)	86.05 (0.537)	0.687 (0.008)
Cat 5: Position-based (20)	87.62 (0.462)	84.67 (1.124)	89.70 (0.314)	0.744 (0.009)
Total (34)	87.20 (0.434)	85.84 (0.790)	88.17 (0.604)	0.737 (0.008)
Top ranked (12)	89.53 (0.480)	89.64 (0.673)	89.46 (0.753)	0.786 (0.009)

The number of features in each category or set is given in parenthesis. Accuracy (ACC), specificity (SPE) and sensitivity (SEN) are expressed as percentages.

**Figure 5:**
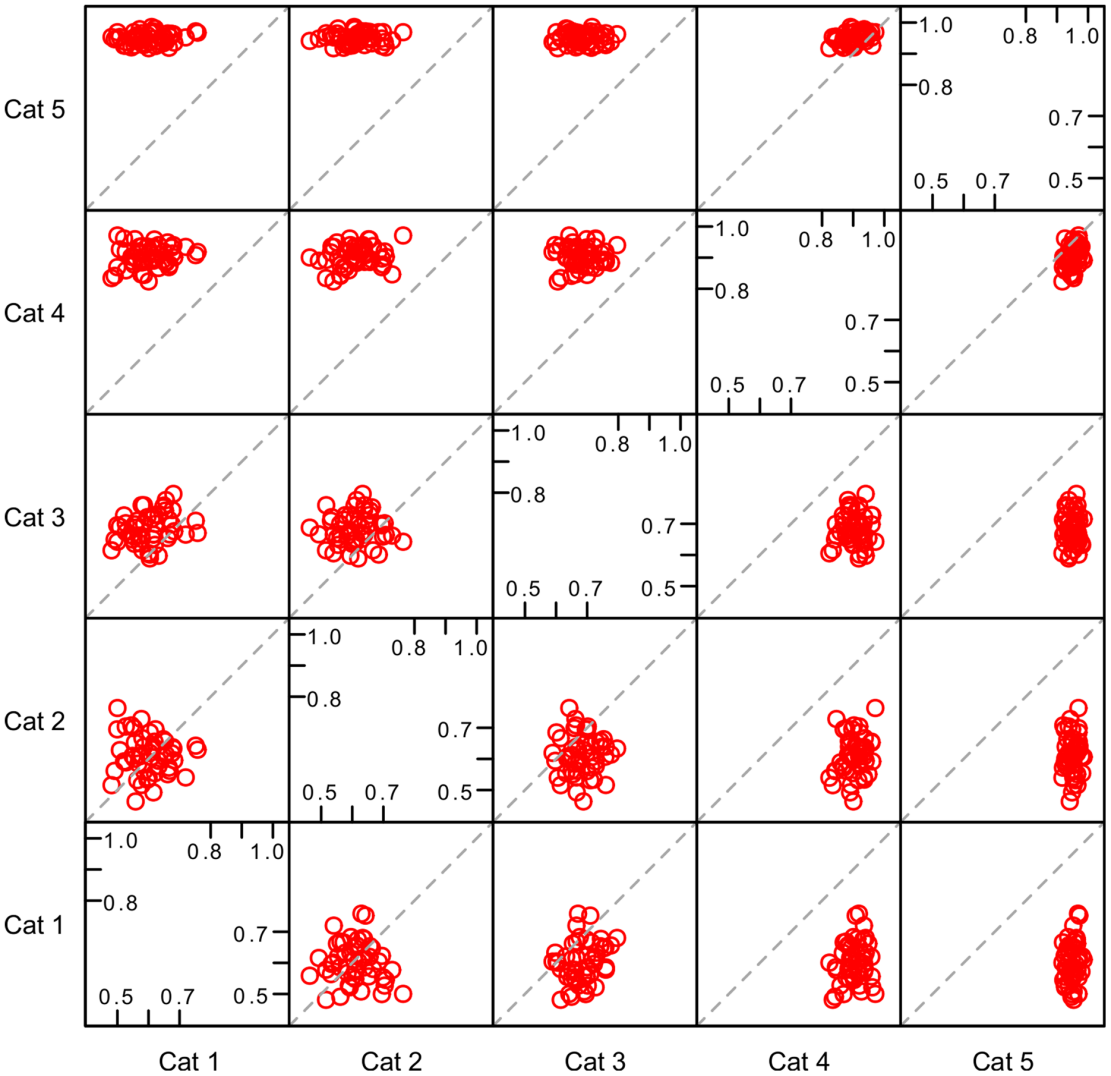
Pairwise correlation between feature categories resampled performance. Points concentrated around the diagonal in the top right corner of plots represent a pair of feature categories with strong relationship and good joint performance, while points widely spread in the bottom left corner of plots depicts the existence of a weak relationship between two categories that also perform weakly in the resampled evaluation. Categories one (alignment features) and two (thermodynamic features) have weak relationship and both have poor resampled performance, whereas categories four (seed features) and five (position-based features) show strong relationship, both of them with high resampled performance.

The analysis of pairwise correlation of the features categories performance based on the resampling approach is shown in Figure 5. Points concentrated in the top right corner around the diagonal represent a pair of feature categories with strong relationship and good joint performance, while points widely spread in the bottom left corner of plots depict the existence of a weak relationship between two categories that also have a poor performance in the evaluation based on resampling. We observe that categories one (alignment features) and two (thermodynamic features) have weak relationship and both have poor resampling performance, whereas categories four (seed features) and five (position-based features) show strong relationship, both of them with high resampling performance. The correlation in performance of categories four and five is in some sense expected since part of the information drawn by seed-related features, namely the base pairing in this region, is also captured by position-based features.

Next, we perform a feature relevance estimation assessing the average decrease in the nodes’ impurity measured by the Gini index during the construction of the decision trees ensemble. This step aims at identifying irrelevant features that may mislead the algorithm and increase the generalization error [36]. Even though RF naturally provide an estimative of feature relevance computed during the course of training, the algorithm lacks a feature selection process: each of its nodes is split based on the optimal choice among a random subset of features. As each decision tree in the ensemble may be regarded as an independent learner trained upon a distinct set of features, the information gain computed during the learning process is not just a good estimation of the individual feature performance, but also of features’ ability in a variety of possible feature subsets [37]. Thus, by estimating the features relevance one can perform a feature selection process to improve the model’s overall performance.

The features ranking in a decreasing order of relevance, measured by the average decrease in the Gini index, is given in Table 4. Our analysis corroborates previous studies in the area [15], [25], [38]: nucleotides surrounding the seed sequence are indeed important for target recognition. Obad and colleagues [38], for instance, discuss a method for antagonizing miRNA function via seed-targeting. They observed the importance of targeting the miRNA seed and suggest that this region is more accessible for miRNA inhibition.

**Table 4:** Features importance.

Rank	Feature name	Mean decreaseGini index
1	MFE of seed region	73.382
2	Position 2	25.282
3	G:C’s in seed region	23.232
4	MFE of complete alignment	20.210
5	Position 4	18.036
6	A:U’s in complete alignment	14.937
7	Alignment score	14.894
8	G:U’s in seed region	14.12
9	A:U’s in seed region	13.23
10	Position 7	12.702
11	Position 6	12.104
12	G:C’s in complete alignment	11.028
13	Position 15	10.043
14	Alignment length	9.954
15	Position 13	9.702
16	Mismatches in complete alignment	9.672
17	Position 3	8.644
18	Position 16	8.576
19	Position 5	8.249
20	Position 8	7.709
21	Position 9	7.667
22	G:U’s in complete alignment	7.297
23	Position 1	6.625
24	Position 10	6.114
25	Position 14	6.024
26	Position 11	5.978
27	Position 20	5.770
28	Position 18	5.348
29	Position 17	5.045
30	Position 12	5.027
31	Gaps in complete alignment	4.895
32	Position 19	4.087
33	Mismatches in seed region	2.939
34	Gaps in seed region	0.000

Ranking given according to features importance computed in the course of training. The decrease in nodes impurity, measured by the Gini index, is computed as the average among all trees.

The analysis of the top ranked features in Table 4 is consistent with the biological knowledge about the relevance of the pairing of the miRNA 5′ region to the mRNA, as it comprises basically properties related to the seed region. Most of the features in the top ten group consist of structural and position-based features regarding nucleotides 2–8, which form the seed region. Furthermore, the seed MFE and number of G:C pairings in the seed region, which correspond to the first and third top features respectively, are known to be important determinants of miRNA-target interaction activity [35].

A consistency is also found for the relevance order concerning Watson-Crick matches, i.e., G:C and A:U, and G:U wobble pairs in the seed region. The highest impact of G:C pairings for target recognition among these is biologically plausible because they are bound by three hydrogen bonds, which makes RNA with high GC-content much more stable than RNA with low GC-content. Thus, G:C pairings in both seed region and total alignment are rated high in the features relevance rank. In contrast, A:U pairings are bond by two hydrogen bonds, justifying the lower stability and position in the features ranking. What was interesting, tough, is that our feature analysis was able to detect the relevance of wobble pairs to miRNA target recognition, which are the most common and highly conserved non-Watson-Crick base pairs in RNA [39]. It was recently found that the thermodynamic stability of a wobble base pair is comparable to that of a Watson-Crick base pair and that they are highly detrimental to miRNA function despite its favourable contribution to RNA:RNA duplexes [35].

### Building a RF Model Based on the Top Ranked Features

Based on the features ranking of Table 4, we perform a restricted forward feature selection: we assess features impact to the model’s predictive accuracy in an incremental fashion and further apply the results for a feature selection process. The first step consists in training several RF models, starting from a single-feature model, and adding each feature at a time from the most relevant to the least relevant. For each of the classifiers generated, we assess their performance computing its accuracy, MCC, specificity and sensitivity for the OOB data. We remind reader that the OOB data is the portion of data not used to grow the decision trees, thus providing an unbiased estimative of performance and overfitting.

**Figure 6:**
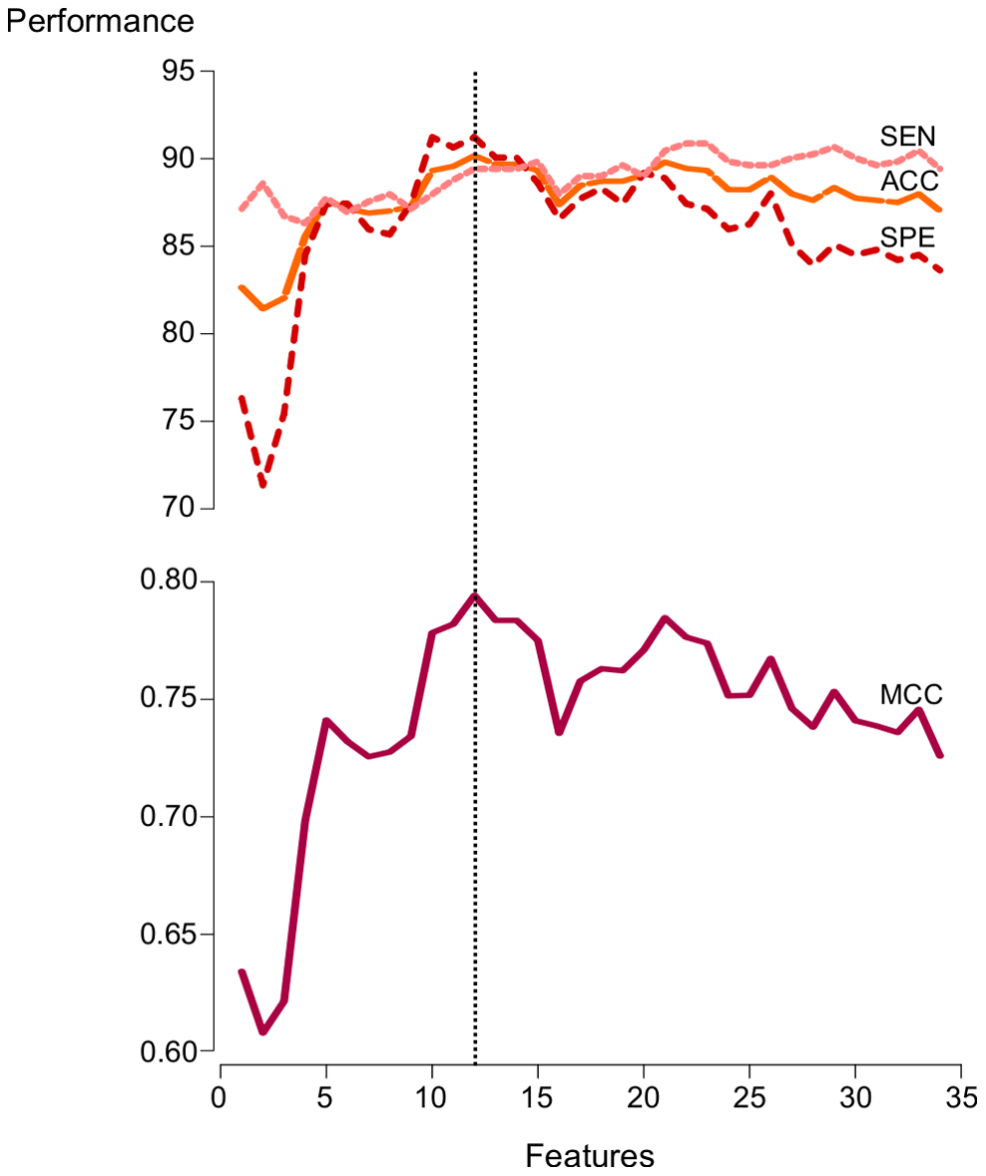
Performance of the RF model evaluated by means of a restricted forward feature selection. Several random forest classifiers were trained adding each of the features at a time, following the rank based on mean decrease of Gini index. The best and most balanced performance in terms of sensitivity and specificity is achieved by the model trained based on the subset of top 12 features.

Results for the restricted forward feature selection are shown in Fig. 6. A peak in the performance can be clearly identified for the model trained upon the set of top 12 features when considering accuracy and MCC scores. Also, one can observe that the use of all 34 features in our training set helps to maintain a model with good sensitivity. On the other hand, it also causes an increase in the generalization error for the negative class, thus impairing the model’s specificity. According to Fig. 6, the best balance between specificity and sensitivity is achieved by the model trained with the 12 most relevant features. Grounded on this observation, we apply a feature selection step by removing the most irrelevant features from the data. Feature selection is known for improving the performance of learning models by enhancing both the generalization capability and the model interpretability. Thus, we repeat the RF training process for a subset of features defined by features 1–12 in Table 4 (the top 12), optimizing the number of variables to choose from in each node split by means of the caret R package.

The results for the top 12 features model are summarized in the confusion matrix of Table 5. Again, these results represent the mean (and standard deviation) computed over five repetitions of 10-fold cross-validation. We observe the robustness of the top 12 features model with respect to the previous model: classification error rates decrease to 10.53% (standard deviation 0.75%) for positive examples and to 10.35% (standard deviation 0.67%) for negative examples, yielding a better and more balanced performance. Moreover, the model’s average specificity and sensitivity are 89.64% and 89.46%, respectively. The better balance between prediction errors for the positive and negative classes is also reflected in the higher MCC, which increased from 0.737 to 0.786. This increase corresponds to about 6% of performance gain over the 34-features RF model, thus evidencing the benefits of performing a feature selection step when training ML classifiers.

**Table 5:** Feature selection improves RFMirTarget performance.

		Real	
		Non-Target	Target
**Predicted**	Non-Target	306.6 (2.30)	50.8 (3.63)
	Target	35.4 (2.30)	431.2 (3.63)

Confusion matrix for the top 12 features RF model estimated by averaging the results over five repetitions of 10-fold cross-validation. Standard deviations are given in parenthesis.

### Further Analysis of the RF Model

In the previous section we have made the point that the top 12 features played an interesting role in the performance of the model, but the overall model’s specificity was marginal due to the misclassification of the negative instances. Classifier methods usually do not effectively handle data correlation and data with imbalanced class. Therefore, in this section we pursue a closer view of strengths and weaknesses of the top 12 features model, while trying to improve its overall accuracy.

Before exploring the data correlation, we evaluate the data scaling issue. It is well-known that traditional classifiers always require a data scaling step before any classification analysis. As an example, SVM classifiers do not perform well without data scaling. Once scaling the data, the overall performance of the model was ACC: 85.29 (0.377), SEN: 87.38 (0.227), SPE: 82.33 (0.842) and MCC: 0.69 (0.008). Although these results are not very competitive regarding the performance of the non-scaled model, we observe that the use of data scaling generates a model able to fit better new, unlabelled instances.

**Figure 7:**
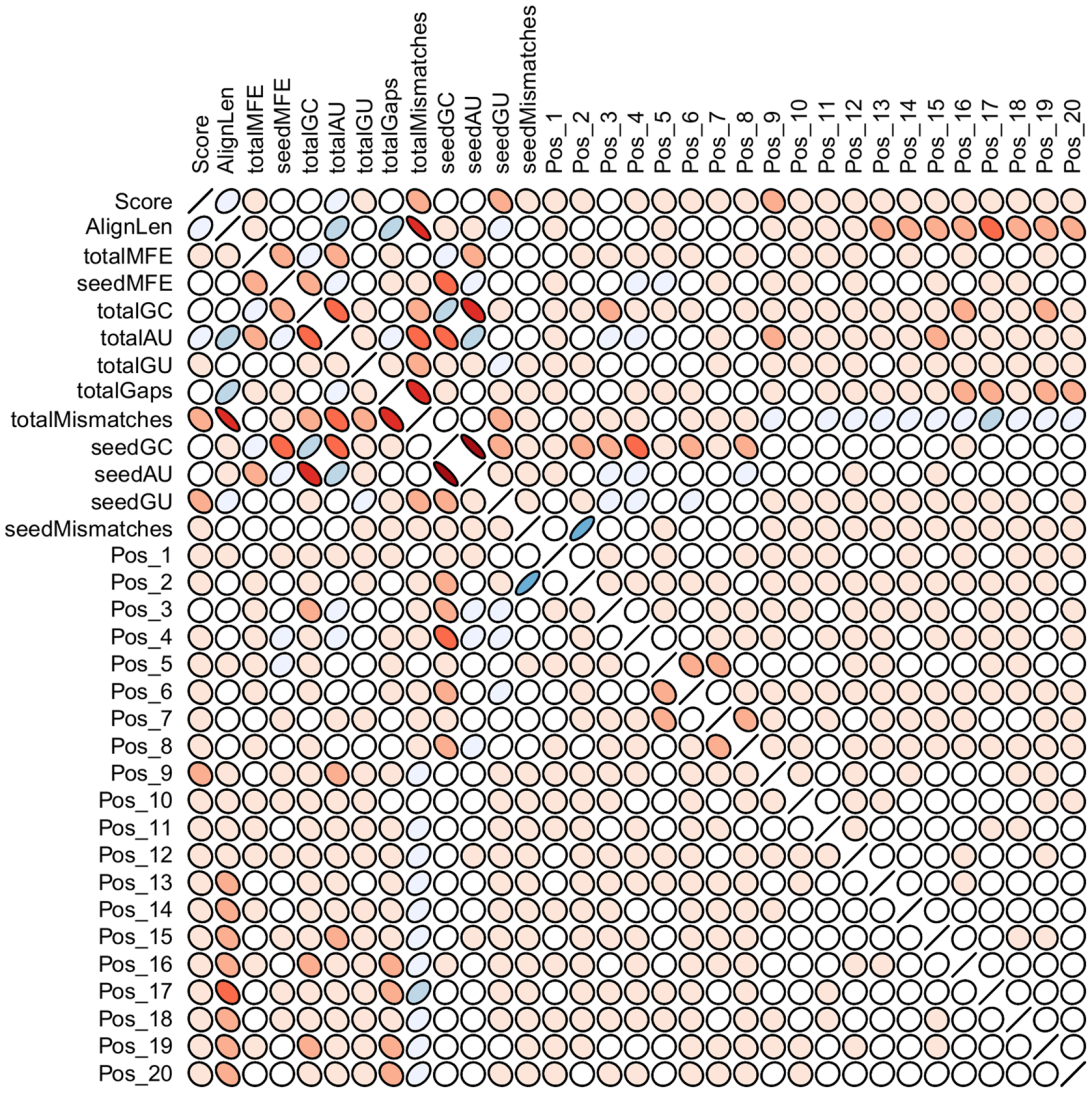
Correlation plot of the 34 features in the training set. Flat circles means strong correlation and regular circles weak correlation. Positive correlation are depicted by blue ellipses bending to the right, while negative correlation is given by red ellipses bending to the left.

**Figure 8:**
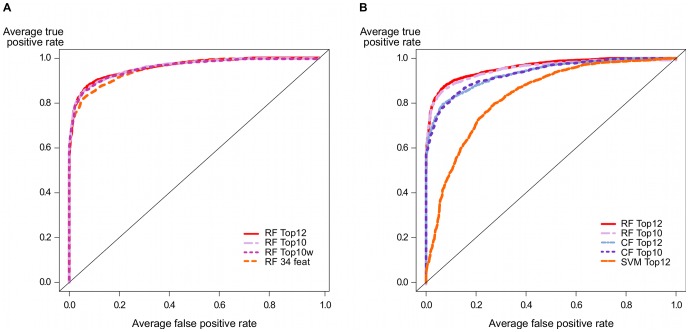
ROC curves comparing several variations of the random forest model. A) The random forest model trained after feature selection (RF Top12) shows a good improvement in relation to the model trained with the total set of features (RF 34 feat). However, some model improvements such as elimination of correlated features (RF Top10) and assignment of class weights (RF Top10w) do not pose difficulty to the performance of the top 12 features model. B) Moreover, we investigate the impacts of conditional permutation in the top 12 features model and the top 10 features model (eliminating strong correlation) and observe that classical permutation still provides a better overall performance. We compare the tree-based models to a SVM classifier trained with the top 12 features, showing that the former achieve a better average performance over all resamples than the latter.

After, we explore data correlation over the total set of features. We note that a few features are strongly correlated (Fig. 7). The seed G:C content, for instance, negatively correlates with the MFE of the seed region and the seed A:U content. In fact, strongly correlated features such as seedAU and totalGC are also in the subset of top 12 features. We evaluate the impact of the elimination of these features and devise a top 10 model. The classification results drawn from repeated cross-validation was ACC: 88.98 (0.621), SEN: 90.04 (0.568), SPE: 87.48 (1.297) and MCC: 0.77 (0.013). Again, the model trained with the data analyzed for features correlation did not pose great challenge to the top 12 model. We also investigate the impact of the imbalanced classes. Differently from the previous model, we set misclassification penalties of 70% and 30% for negative and positive classes, respectively. By taking into account such model weighting we are able to achieve a top 10 weighted model that handles more effectively the identification of class imbalance issue. We compare these models in terms of the ROC curves (Fig. 8A), observing that the elimination of correlated features and the misclassification penalties show a slight performance improvement in relation to the 34 features model, but still not enough to outperform the top 12 features model.

It is also known that the permutation importance of RF models is based on a random permutation of the predictor variables. The Gini coefficient has been shown to carry forward the bias of the underlying Gini-gain splitting criterion when predictor variables vary in their number of categories or scale of measurement. Though it is a practical solution of several classification tree methods, a conditional permutation scheme would be more suitable, preserving the correlation structure between target and predictor variables. We investigate the impacts of conditional permutation by exploring two additional models and observe that classical permutation with a weighting scheme does provide a good overall performance (see Fig. 8B), but without outperforming the classical random permutation. However, we understand that whether a marginal or conditional importance model is to be preferred depends on the research question under investigation.

### Comparison with Other Classifiers

In order to perform a more thorough evaluation of our top 12 RF classifier, we compare it against several popular classifiers in the ML field trained with the same set of features, some of which were already applied to the problem of predicting miRNA target genes: i) J48, an open source Java implementation of the C4.5 algorithm for building decision trees; ii) Naïve Bayes (NB), a statistical classifier used in the development of NBMirTar [21]); iii) k-nearest neighbors (KNN), an instance-based learner; iv) SVM, a classifier used as basis in most of the current available ML-based methods for the prediction of miRNAs targets, e.g., miTarget [22], TargetMiner [23] and MultiMiTar [24]); and v) GLM, a generalized linear model. For such comparison, we use the caret R package and perform a repeated 10-fold cross-validation, averaging results over five repetitions. In addition, as different classifiers require different levels of parameter tuning, we also adopt the caret package interface for training functions in order to optimize particular parameters of each of the counterpart classifiers.

**Figure 9:**
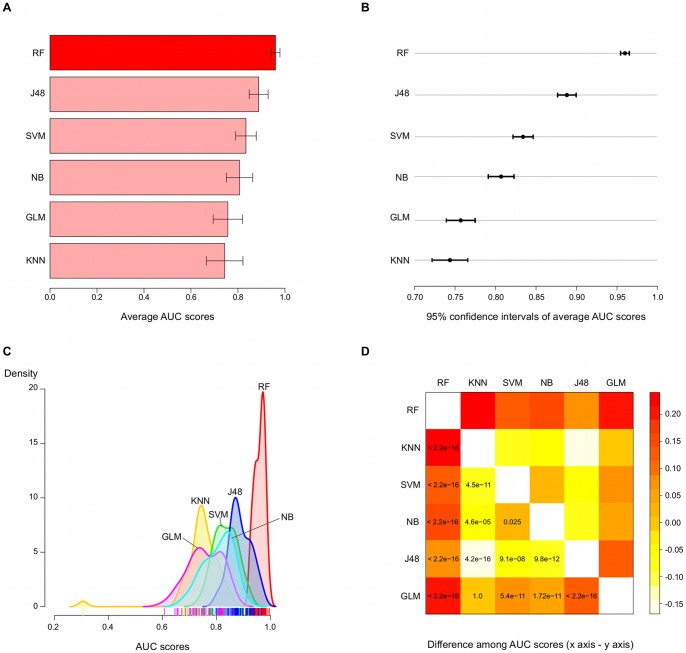
Comparison of our random forest model against several popular classifiers based on repeated cross-validation. We compare the top 12 features RF model with five others popular classifiers trained with the same features set: J48, K-nearest neighbours (KNN), SVM, Naïve Bayes (NB) and a generalised linear model (GLM). A) The average AUC score, computed as the mean over five repetitions of 10-fold cross-validation, is greater for the RF model, which also presents the smallest standard deviation among all classifiers. B) A comparison of average AUC scores in terms of 95% confidence intervals evidences the statistically significant superiority of the RF model. J48 also shows a significant difference in performance regarding the remaining methods, but still lower than RF’s. C) Moreover, the performance of the classifiers over several resamples are summarised by a kernel density estimator, which indicates a tall and narrow distribution for our RF classifier. This gives a picture on the robustness of the RF model: our classifier is not only better (distribution is shifted towards upper limit of x axis, i.e., highest scores), but also shows a more consistent performance (narrower distribution in relation to others). D) Finally, a t-test over pairwise differences in average AUC scores across all classifiers produces very small p-values (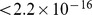) for comparisons against the RF model, providing additional support to the superior performance of the proposed method.

Results for this comparative analysis are shown in Fig. 9. The average AUC scores, computed as the mean of the area under the ROC curves over all repetitions of cross-validation, is around 0.96 for RF model, in contrast to 0.89 for the second best performing classifier, J48 (Fig. 9-A). This represents a performance gain of almost 8%, which is shown to be a significant increase based on the analysis of 95% confidence intervals of average AUC scores (Fig. 9-B). In fact, 95% confidence intervals reveal the statistically significant performance superiority of RF model in relation to all other classifiers.

Moreover, densities plot of AUC scores based on the resamples depict the robustness of RF model. The proposed model has its density distribution shifted to the right of x-axis (highest scores) (Fig. 9-C), with a much more narrow shape when compared to counterpart methods, meaning a better and more consistent performance. Finally, we perform a pairwise t-test comparing the RF model against each of its counterpart methods in terms of difference in average AUC scores (Fig. 9-D). The statistical test produced very small p-values (p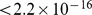) for all of the carried comparisons, indicating that the performance of the RF is significantly superior in relation to the remainder algorithms. Therefore, the outcome of the classifiers comparison supports the better performance of the RF algorithm in contrast to commonly applied ML methods, as well as the good potential of our tool in predicting new miRNAs target genes. One reason for such improvement might be associated to the robustness of the RF algorithm to the class imbalance problem, which usually impairs the performance of competing classifiers such as SVM.

### Evaluation on Completely Independent Test Data

To further assess the predictive power of the proposed RF classifier and strengthen our comparative analysis, we download a collection of 172 experimentally supported human miRNA targets and 33 experimentally confirmed false target predictions from the TarBase 5.0 [40] to serve as an independent test data set. The performance of RFMirTarget is compared to the counterpart methods outlined in the previous section for both the complete set of features and the subset of top 12 features (see Table 1).

**Figure 10:**
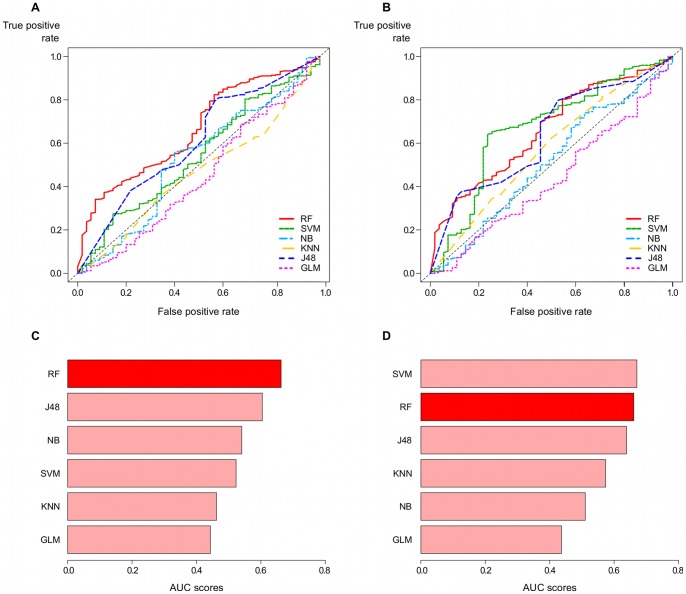
Comparative performance of RFMirTarget for a completely independent test data set. We test the proposed RF model with a collection of experimentally verified positive and negative examples downloaded from TarBase 5.0, comparing it against some counterpart methods. Panels A and C refer to models trained with the complete set of features, whereas panels B and D present results for the training process based on the subset of top 12 features. This analysis raises more evidence to the good overall performance of our method in relation to other popular classifiers. In general, ROC curves for the RF model (panels A and C) are more far away from the diagonal line, which denotes a random classifier with no predictive power. Moreover, a permutation test based on label randomisation to evaluate the statistical significance of the computed AUC scores returns the lowest p-values for the RF-based approach in both situations (training with all features and training with the top 12 features), indicating that these results would be unlikely to occur by chance.

Results in terms of ROC curves and AUC scores are shown in Fig. 10. Panels A and C depict the performance for models trained with all features, while panels B and D show the results for the top 12 features models. Furthermore, ROC curves for all classifiers considered are shown in top panels, whereas the computed AUC scores are compared in the bottom panels. These plots show that the RF and J48 models present the best performance when considering the complete set of features, as their ROC curves have the greatest distance from the dashed diagonal line, which represents the performance of a random classifier (Fig. 10A and Fig. 10B). In contrast, KNN and GLM perform as poor as a random classifier.

However, when focusing the training process solely in the most relevant features, i.e., the top 12 set, SVM and KNN show an important boost in their predictive accuracy. In fact, SVM outperforms the RF classifier for the top 12 features models, obtaining higher true positive rates for false positive rates in the approximate range of 0.2 to 0.6. A comparison in terms of the AUC scores (Fig. 10C and Fig. 10D) summarise these results in a more straightforward fashion. We observe that both RF models outperform all other classifiers but the SVM model trained on the set of most relevant features. In addition, one can clearly notice the changes in the classifiers performance ranking when switching from the total set of features to the subset of top 12 features: KNN and SVM, in particular, rank higher in the latter.

To assess the statistical significance of the AUC scores shown in Fig. 10, we perform a permutation test. Given the original labels (classes) of the test data set, we permute its values to obtain a randomized version of the labels and then reevaluate the prediction accuracy for each of the models compared. We repeat this process 2000 times and compute a p-value, which represents the fraction of randomized samples in which the classifier performs better than in the original data, and indicates how likely the observed accuracy, e.g. the computed AUC scores, would be obtained by chance. Very low p-values (p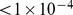) are obtained for both RF models, giving additional evidence for the good performance and robustness of our proposed classifier, even when considering an independent test set. In addition, J48 has p-values p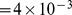 and p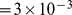 for the 34 features and top 12 features models, respectively, while SVM only shows statistical significant performance for the top 12 features version (p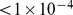). All the remainder models do not pass the statistical significance test (p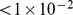).

When comparing the class probabilities assigned by each of the algorithms trained with the complete set of features (Fig. S1 and Fig. S2) we verify a large overlap between the misclassified instances. The proposed RF model presents very few classification mistakes, especially for the positive class (Target), and for most of the instances misclassified by our model, the counterpart methods also show difficulty in predicting the correct class. Since the training data set is a common factor among all classifiers, this observation might suggest a methodological bias in the data set rather than issues such as overfitting, yielding a poor generalization ability over new instances defined under different validation protocols. At this point, we remind reader about the methodology adopted by Bandyopadhyay and Mitra [23] in the formulation of the training data set, which involves the use of expression data to determine real and pseudo miRNAs targets. As we shall discuss later, this procedure might bias the training data towards a subset of miRNAs that act by specific targeting mechanisms.

**Figure 11:**
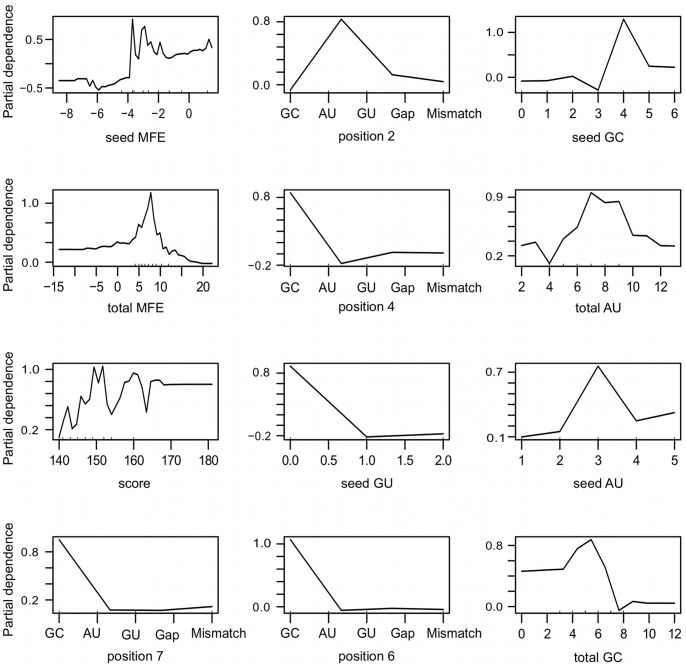
Partial dependence plots for the top 12 relevant features. These plots depict the effect of different values of a given variable on the class prediction, allowing us to further interpret the ensemble model and its weaknesses. Structural features related to the seed region, namely the absolute frequency of G:C, A:U and G:U alignments, show an unimodal curve, with very well defined values for high class probabilities. One can observe, for instance, that three or more G:C alignments in the seed region may disrupt correct classification of true miRNA-target pairs, while three A:U alignments is very decisive for the recognition of true positive instances. Moreover, as expected, G:U occurrence drastically decrease the class probability. In what concerns the position-based features, the analysis of the plots suggest that a G:C alignment in position 2 in detrimental for miRNA-target recognition, conversely to what is observed for positions 4, 6 and 7.

To better understand the misclassifications of our model for the TarBase independent test set and further explore the features relevance on classification results, we generate partial dependence plots for the top 12 relevant features as shown in Fig. 11. Partial dependence plots give a visual picture about the marginal effect of a feature on the class prediction. In other words, for each value a feature may assume, the plot estimates the approximate chance of an instance being classed as a true miRNA-target pair. One can observe that the occurrence of a wobble (G:U alignment) in positions 4, 7 and 6 of the miRNA-target alignment has a negative effect over the class probability, whereas in position 2 it yields higher chance of correct classification. Moreover, a G:C alignment in position 2 seems to disrupt the classifier’s predictive accuracy. In fact, from the 43 misclassified positive instances of the independent test set (the false negatives), 51% show a G:C alignment in position 2, what might partially justify the incorrect classification. In what concerns the structural features related to the seed region, namely the absolute frequency of G:C, A:U and G:U alignments, the partial dependence plots show an unimodal curve, with very well defined values for high class probabilities. We found that about half of the positive instances that were misclassified by RFMirTarget have three or less G:C alignments in the seed region, which are known to be important for RNA stability and miRNA binding and might thus mislead classification. Furthermore, we verify that only about 30% of the false negative instances have favourable absolute frequency of A:U pairing in the seed region and that the relative frequency of wobbles in the seed region of false negatives is significantly greater that what we observe for the true positive examples (p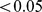, Fisher’s exact test).

**Figure 12:**
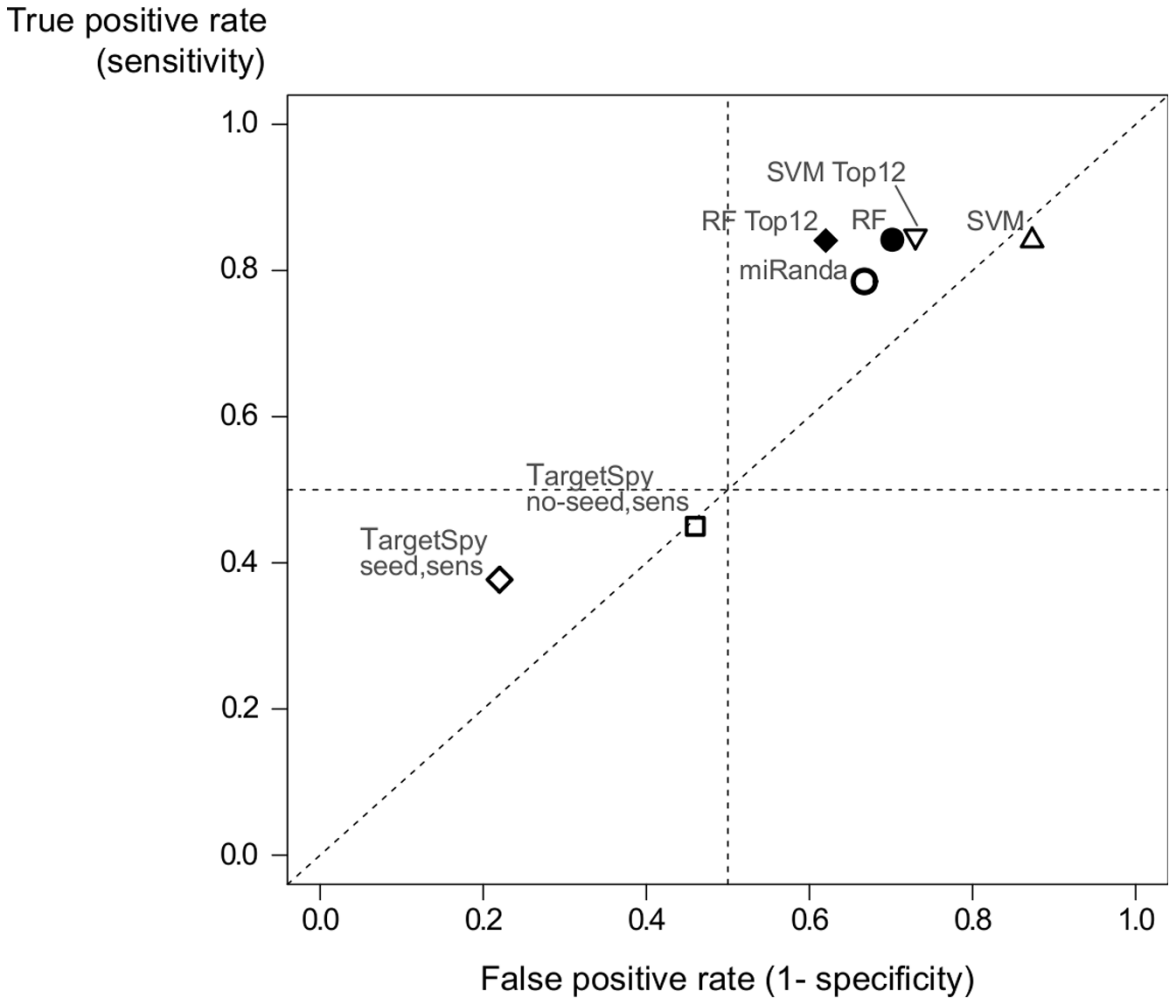
Comparison of false positive and true positive rates for several distinct methods based on an independent test set. Our RF model has a very good overall performance compared to miRanda and TargetSpy, two other algorithms for miRNA target prediction, as well as to a SVM classifier trained with the same features and data set. Its sensitivity it’s among the highest, and it is also the farthest from a random performance, denoted by the diagonal line. However, the high sensitivity comes at the cost of increased false positive rates. In this sense, TargetSpy is the most reliable tool among the methods compared in terms of correct identification of false targets. As usual, there is a clear trade-off between true positives and false positives, and the decision of which classifier to apply ends up depending on the specific application and to which extent the occurrence of false positives are accepted.

Next, we compare our RF classifier against other target prediction algorithms, miRanda and TargetSpy. While miRanda predicts targets mostly upon sequence complementarity miRNA-target duplex thermodynamics, TargetSpy is a ML approach that applies feature selection and a learning scheme based on boosting with decision stumps as base learner. For TargetSpy, we run two versions of the algorithm, one with seed match requirement (*TargetSpy seed sens*) and the other without seed match requirement (*TargetSpy no-seed sens*), both using the sensibility as the threshold score [11]. Based on the confusion matrix built from each of these methods predictions for the independent test data set, we compute their specificity and sensitivity. Results are shown in Fig. 12, which plots the false positive rate (1-specificity) versus the true positive rate (sensitivity) for several methods, including our RF model and a SVM model trained with our set of descriptive features.

Two things to be noted about Fig. 12 is how far points are from the dashed diagonal line, which denotes a totally random method without any predictive power, and in which quadrant points are situated. Ideally, one would expect methods whose points are located in the top left quadrant of the plot, meaning high sensitivity and high specificity, and far away from the diagonal line. However, in the comparison carried in this paper, none of the algorithms achieved such desirable performance. Our RF classifier, in particular the top 12 features RF model, is shown to have a sensitivity higher than miRanda and TargetSpy, and is also plotted further away from the diagonal line in relation to other methods.

Although SVM reaches a sensitivity very close to our model’s, it has a lower specificity, degrading its overall performance. In fact, in what concerns the specificity, the proposed RF models perform weaker than the two variations of TargetSpy, which achieves very low false positive rates. On the other hand, TargetSpy also has the lowest true positive rate among all algorithms: only about 37% to 45% of true targets are correctly identified. Therefore, the proposed RF models are reliable in the sense of identifying a higher number of true positive targets, due to its outstanding sensitivity, but at the cost of increased false positive rates. Under the best of circumstances, one wishes a classifier with a perfect balance between sensitivity and specificity. However, in most cases accuracy is still constrained by the trade-off between true positives and false positives, and the decision of which classifier to apply depends on the specific application and to which extent the occurrence of false positives are tolerated [41].

### Estimating the Prediction Accuracy on CLIP-Seq Data

To conclude our comparison using independent data, we gather two new data sets from the starBase platform (http://starbase.sysu.edu.cn) [42] regarding CLIP-Seq (cross-linking immunoprecipitation-high-throughput sequencing) data containing true miRNA-target interactions and test the accuracy of our method in the identification of positive instances, i.e., its sensitivity. In general, real and pseudo miRNA-target interactions available in databases such as TarBase are based on bioinformatics predictions, and most of the softwares used to predict miRNA-target interaction sites have high false positive rates. Due to both the short length of miRNAs and to the imperfect base-pairing, many possible miRNA-target interaction sites can be identified throughout the transcriptome for a single miRNA, but just a few of these are indeed functional. In order to determine biologically relevant miRNA-target interaction sites, the high-throughput sequencing of RNA isolated by cross-linking immunoprecipitation of Argonaute (Ago) protein has been used [43]–[45]. This approach restricts the number of possible miRNA binding sites to those that are found physically bound to an Ago protein, thus they are more likely to be functional. Several studies show that the application of this method has significantly reduced the rate of false positive predictions of miRNA-target interaction sites [42]–[45], thus representing a high-quality and reliable data to test the performance of computational approaches.

Using the tool *target site intersection* of the starBase platform, we search for miRNA-target interactions involving any of the human miRNAs available at starBase that are simultaneously predicted by at least four softwares (TargetScan, PicTar, RNA22 and PITA). Moreover, we adopt the most restrict value for the minimum number of reads (1000 reads) and require a biological complexity (BC) equal or higher than 2. In this analysis, 385 miRNA-target pairs are found. To avoid overfitting, if more than one miRNA with the same predicted target site is found for a given gene, we randomly select one of the possible miRNAs and exclude the others from the data set. Further, we divide the data in two different data sets: (i) one containing the miRNA-target pairs that were not predicted by miRanda (38 pairs) and (ii) one containing miRNA-target pairs predicted by the four aforementioned softwares and also by miRanda (170 pairs).

Results for the CLIP-seq data are shown in Table 6 and compare the sensitivity for the six in-house trained classifiers, as well as the predictions by the TargetSpy software. For the latter, we adopt the sensitivity as the threshold and run both variants of the algorithm, with and without the seed requirement. All instances for which the predicted probability is higher than 0.5 are classified as Targets (the true positive instances). Similarly to what we observe in tests with the TarBase data, TargetSpy achieves very low sensitivity levels for both data sets. In its best performance (run with no seed requirement for data set #2), TargetSpy recovers only about 53% of the positive examples. This finding confirms that despite the low false positive rates returned by TargetSpy in the tests with the TarBase data, this tool is not very efficient in the identification of real miRNAs target genes.

**Table 6:** Comparison of methods’ sensitivity for tests performed with the CLIP-Seq data.

Method	Features/Setup	Data set #1	Data set #2
RF	complete set	0.704	0.725
	top 12	0.774	0.756
SVM	complete set	0.464	0.549
	top 12	0.901	0.891
NB	complete set	0.591	0.657
	top 12	0.633	0.689
KNN	complete set	0.661	0.756
	top 12	0.675	0.633
J48	complete set	0.521	0.586
	top 12	0.732	0.743
GLM	complete set	0.000	0.027
	top 12	0.000	0.018
TargetSpy	seed	0.421	0.339
	no-seed	0.459	0.529

Data set #1 refers to interactions predicted by all softwares except miRanda (38 pairs).

Data set #2 comprises interactions predicted by all softwares, including miRanda (170 pairs).

Both TargetSpy tests were performed using the sensitivity as the threshold.

In contrast, classifiers trained with our defined set of features achieve much higher accuracy. Except for the GLM classifier, which fails in this test, most of classifiers predictive accuracies outperform TargetSpy, especially when feature selection is applied (top 12 features). Moreover, as opposed to what one would expect, there is no bias in the performance regarding the data set built upon evidence from miRanda (data set #2), as in some cases classifiers perform better for interactions that were not predicted by miRanda than those that are supported by miRanda. Our RF classifier trained with the complete set of features presents a sensitivity that ranges from 70.4% to 72.5%. In this scenario, the only classifier that outperforms our tool is the KNN, which correctly classifies 75.6% of the instances from data set #2.

One interesting observation regarding values in Table 6 refers to the impact of feature selection over results. We observe that RF, SVM and J48 especially benefit from a feature selection process. The proposed RF model succeeds in identifying up to 77% of instances with a low complex model, trained over 12 features, presenting a performance gain of 9.94% for data set #1 and 4.27% for data set #2. The J48 classifier, which builds a single decision tree, has a much higher improvement in performance, increasing its sensitivity in 40.49% and 26.79% for data set #1 and data set #2, respectively. Moreover, the sensitivity achieved by SVM after feature selection is surprisingly high. The classifier correctly identifies about 90% of the true miRNA-target interactions for both data sets, highlighting the importance of feature selection in the SVM’s learning convergence and generalization performance. In contrast, RF is very robust to these factors and able to perform satisfactorily well with much less setup efforts.

Despite the higher predictive accuracy provided by SVM over RF, the analysis of the raw class probabilities assigned by both methods reveals that SVM tends to produce lower probabilities for both data sets tested, conversely to what is observed for RF, which in general has a distribution skewed towards high probabilities (Fig. S3). We compare the mean and median between both methods and conclude that regardless the scenario in terms of CLIP-seq data set tested and number of features used for training, RF always produce probabilities with higher mean and median. For data set #1, the mean (median) are 0.600 (0.608) for the RF model and 0.483 (0.477) for the SVM model, and after feature selection values increase to 0.631 (0.660) for the RF model and 0.576 (0.570) for the SVM model. For tests with data set #2, the mean (median) for the 34-features models are 0.590 (0.594) for RF and 0.530 (0.523) for the SVM, while the values for the 12-features models are 0.609 (0.636) for RF and 0.576 (0.571) for SVM. We compare the probabilities vectors between both methods and find a statistical significant difference (p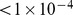, Mann-Whitney test) for every possible scenario described above, confirming the observation that probabilities assigned by our RF model tend to be higher, as one wishes in order to increase the chances of a satisfactory predictive accuracy. In fact, we test the effects of changing the classification threshold to 0.6 and we observe that the proposed RF model conserves a good performance, still correctly classifying around 60% of the instances for both data sets. On the other hand, the performance of the SVM classifier drastically drops, recovering only 30% and 22% of the instances for data sets #1 and #2, respectively, in the best scenario, i.e., under feature selection. Therefore, the proposed model is shown to be more reliable and robust for the prediction of miRNAs target genes when compared to other well-known machine learning algorithm, as well as to popular tools such as TargetSpy.

### Conclusion

The discovery of miRNAs target genes is a crucial step towards the elucidation of mechanisms involved in gene regulation. The important role played by miRNAs in animal development and physiology is well-established. Their participation in metabolic processes such as growth, apoptosis, cell proliferation and stress responses has already been characterized [4], [5], as well as their involvement in several ways in cancer progression [6]. Therefore, increasing efforts have been observed for the development of computational tools aiming at the identification of novel mIRNAs targets.

In the current paper, we discussed a ML approach based on ensemble of decision trees predictions, named RFMirTarget. The choice of the algorithm is motivated by its outstanding performance in other classification problems, including the prediction of novel miRNAs [9]. Nonetheless, few other applications proposed so far for the identification of miRNAs targets have explored this ensemble classification approach. Our experiments have shown that RF indeed performs well in this classification task, being a promising computational approach for miRNA-target prediction. After carrying a thorough analysis of our RF model predictive accuracy, comparing it against several popular classifiers trained with the same data by means of repeated cross-validation, we concluded that RFMirTarget performance is robust and superior to competing methods with statistical significance, with the benefit of requiring much less setup efforts to reach satisfactory performance levels. We show that factors such as data scaling, class imbalance and features correlation do not pose difficulty to the good performance of RFMirTarget as it is usually the case with other classifiers. In addition, the comparative study performed in this work adds to the field in the sense of providing guidance in the choice of the algorithm when it comes to prediction of miRNAs target genes. To the best of our knowledge, a fair and comprehensive comparison of machine learning algorithms applied to this specific task has been poorly addressed in literature.

Moreover, the analysis of features relevance has shown good consistency with important biological properties for miRNA-target alignment stability and also corroborates previous studies in the field that discuss, for instance, the importance of seed region in miRNA-target recognition [15], [25]. In addition, a restricted forward feature selection suggests that the model built upon the subset of top 12 features presents the most balanced classification results in terms of specificity and sensitivity. Results achieved after feature selection are robust and very satisfactory for the majority of the classifiers tested. This shows that the good performance achieved by RFMirTarget is not only due to the classifier chosen, but also to the set of features defined. An interesting point to be observed is that in contrast to what is usually observed in literature, we refine the set of features and devise a low-complexity model that performs reliably well in the desired task based on a small set of 12 features. Counterpart ML-based methods tend to perform training over a much larger set of features, which can compromise the generalization performance of classifiers [36].

Finally, we compared our method’s performance with other tools for miRNA-target prediction, namely TargetSpy and miRanda, as well as counterpart ML algorithms, using completely independent test data sets downloaded from TarBase [40] and starBase [42] platforms. We observed a good overall performance associated with a very small p-value computed based on a label permutation test, suggesting that the performance is not random, but rather statistical significant. In general, RFMirTarget presents the best sensitivity among the tools tested, with a very reliable performance when compared to other methods. Therefore, a direct application of our tool would be to refine results from miRanda, which is used in our framework. However, we emphasize that any other software that provides the predicted sites of alignment between a miRNA and its candidate targets could be use in the place of miRanda, e.g. TargetSpy [11], TargetScan [18], PicTar [19], PITA [46], among others. In fact, it would be interesting to investigate the impact of the aforementioned tools in the classification results, estimating the lower and upper bounds on the performance provided by each of the tools.

Despite the great potential of our tool in identifying true positive miRNA-targets, evaluation based on the TarBase independent test data suggests that it still needs improvement regarding its specificity. The higher false positive rate of RFMirTarget in contrast to TargetSpy in the tests with TarBase data can be related to some extent to the definition of negative examples used as training data set. It is already know that negative examples are harder to obtain than positive ones, and the procedure used in their identification can somehow bias the negative set towards some specific type of miRNAs targeting. For instance, using gene expression in the identification of negative examples might limit the negative set to miRNAs that act by cleavage, whereas some miRNA targeting occurs predominantly at the level of translational repression [47]. Thus, for future work, we deem interesting to evaluate performance of RFMirTarget with data sets derived from protein abundance experiments, as well as expand our training data set by including new examples defined based on distinct validation protocols, for instance, CLIP-Seq data.

The challenge of predicting miRNA target genes is far from being completely solved. Although a plethora of methods have been proposed, most of them take into account several premises such as high complementarity between miRNA and mRNA and the idea of one miRNA to one mRNA interaction. However, as experimentally observed, miRNAs target multiples genes and genes are targeted by multiple miRNAs [48]. Moreover, even in the case of high complementarity, effective target site might not happen due to mRNA accessibility in terms of secondary structure, for instance. Although RFMirTarget presents a promising strategy for Human miRNA target prediction and a reliable source to reduce the set of hypothesis to be experimentally tested, as its counterpart methods, is still not able to effectively handle the previously mentioned issues, a situation that could be of significant computational and biological importance to pursue in near future.

## Supporting Information

Figure S1 **Predicted probabilities for 50 random positive instances of the TarBase independent test set.** The heat map shows the predicted class probabilities by the distinct machine learning algorithms compared when trained over the complete set of features. For positive instances, probabilities higher than 0.5 yield the correct classification (Target). We observe a great overlap of misclassified instances among the algorithms. In general, positive instances not identified by our RF model are also assigned low class probabilities by the counterpart methods, suggesting that errors in classification of independent test instances might be due to artefacts of training data rather than issues such as model overfitting.
(TIF)

Figure S2 **Predicted probabilities for 30 random negative instances of the TarBase independent test set.** For negative instances, probabilities equal or less than 0.5 yield the correct classification (Non Target). We observe that many of the predicted probabilities are situated around the boundary condition that distinguishes the positive class from the negative class, regardless of the algorithm considered. Thus, the compared algorithms show a deficiency in the generalization power concerning the negative class, which could be overcome by enhancing the training data set with more negative examples.
(TIF)

Figure S3
**Density distributions of the class probabilities predicted by RF and SVM models for the CLIP-Seq data.** Panels A and C refer to the tests with data set #1, while panels B and D refer to results related to data set #2. Moreover, the top panels (A and B) are for models trained with the complete set of feature, whereas bottom panels (C and D) are for models trained with the top 12 features. We observe that regardless of the data set used, the distribution of probabilities predicted by RF is skewed to the right, meaning that they tend to be higher than the probabilities returned by SVM. We compare the raw probabilities in terms of a Mann-Whitney test and find a significant difference (p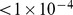) for all the possible scenarios (panels A–D).(TIF)
