# Supplementary figures and images for: RFMirTarget: Predicting Human MicroRNA Target Genes with a Random Forest Classifier
Source: PLoS One. 2013 Jul 26;8(7):e70153. doi: 10.1371/journal.pone.0070153 (PMC3724815; doi:10.1371/journal.pone.0070153)

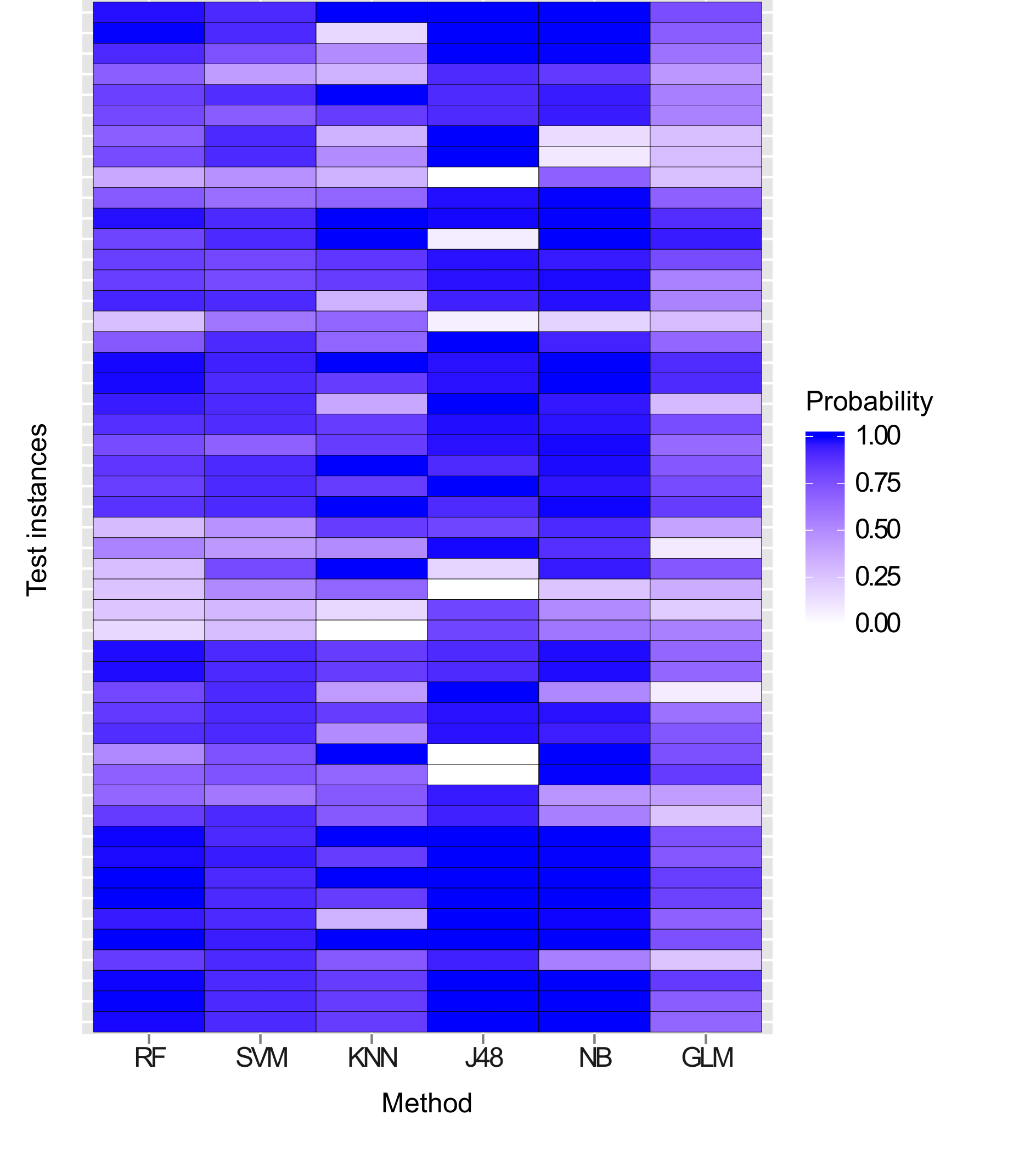

Supplement: Figure S1 — Predicted probabilities for 50 random positive instances of the TarBase independent test set. The heat map shows the predicted class probabilities by the distinct machine learning algorithms compared when trained over the complete set of features. For positive instances, probabilities higher than 0.5 yield the correct classification (Target). We observe a great overlap of misclassified instances among the algorithms. In general, positive instances not identified by our RF model are also assigned low class probabilities by the counterpart methods, suggesting that errors in classification of independent test instances might be due to artefacts of training data rather than issues such as model overfitting. (TIF) [file pone.0070153.s001.tif]

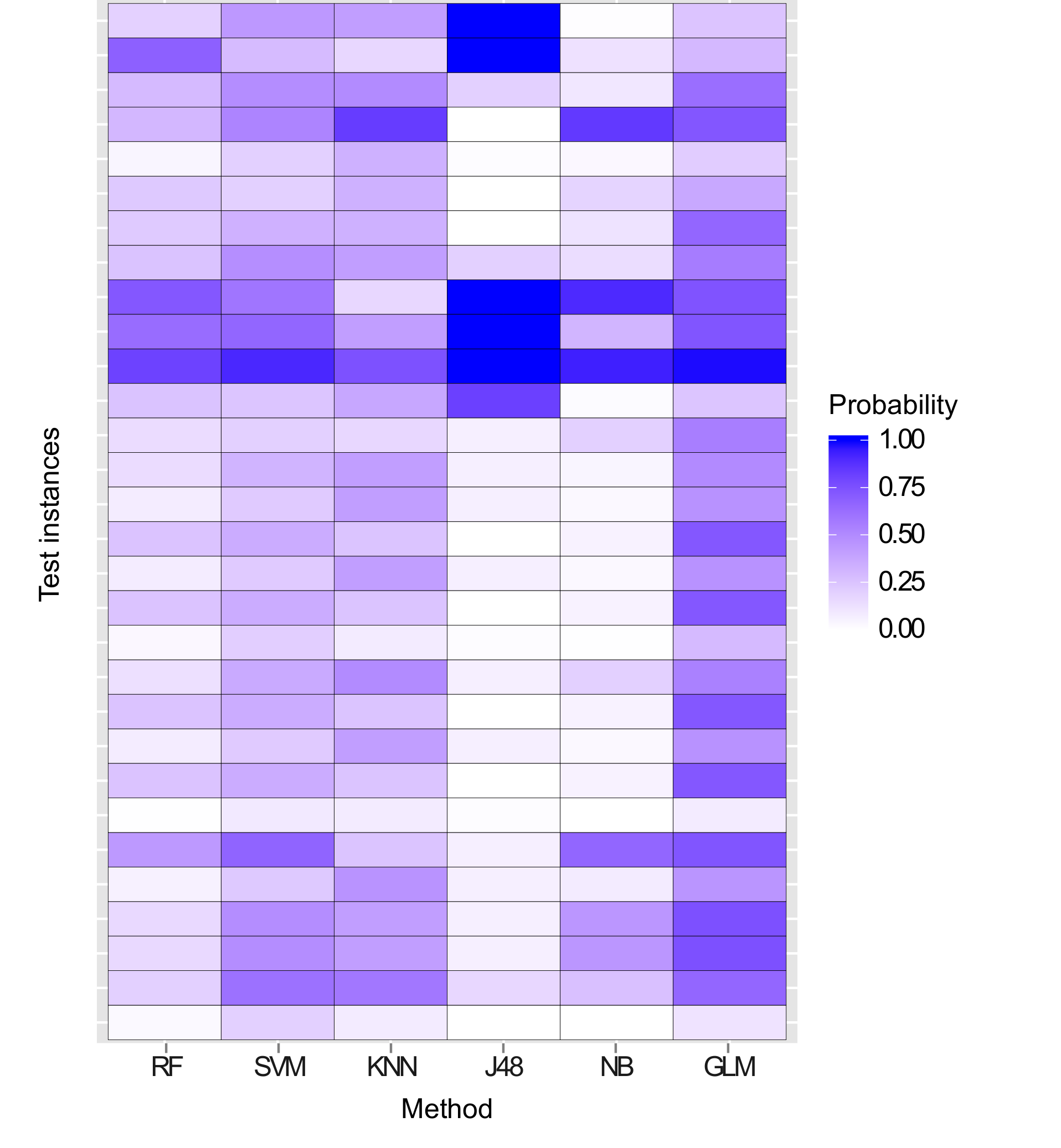

Supplement: Figure S2 — Predicted probabilities for 30 random negative instances of the TarBase independent test set. For negative instances, probabilities equal or less than 0.5 yield the correct classification (Non Target). We observe that many of the predicted probabilities are situated around the boundary condition that distinguishes the positive class from the negative class, regardless of the algorithm considered. Thus, the compared algorithms show a deficiency in the generalization power concerning the negative class, which could be overcome by enhancing the training data set with more negative examples. (TIF) [file pone.0070153.s002.tif]

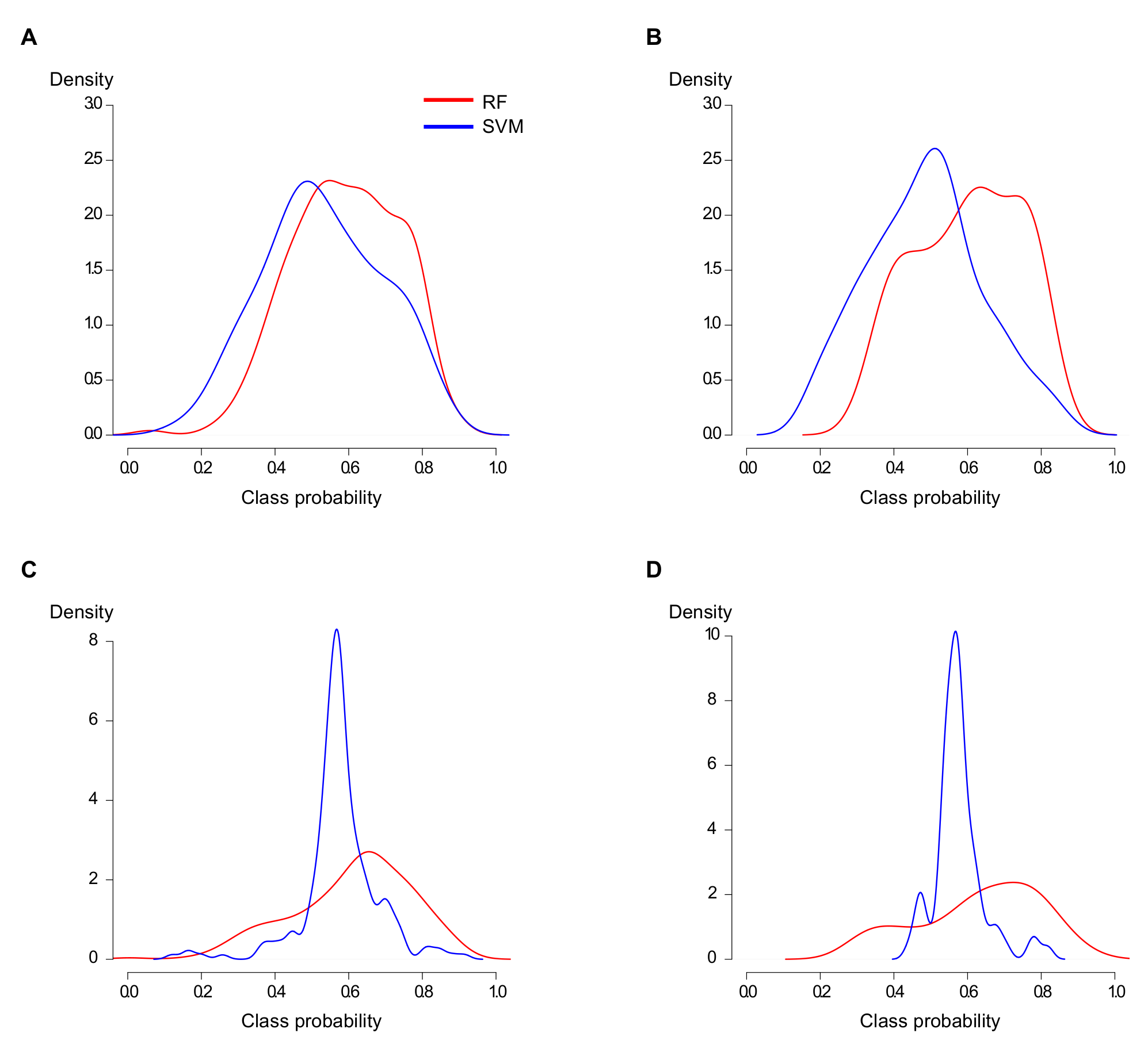

Supplement: Figure S3 — Density distributions of the class probabilities predicted by RF and SVM models for the CLIP-Seq data. Panels A and C refer to the tests with data set #1, while panels B and D refer to results related to data set #2. Moreover, the top panels (A and B) are for models trained with the complete set of feature, whereas bottom panels (C and D) are for models trained with the top 12 features. We observe that regardless of the data set used, the distribution of probabilities predicted by RF is skewed to the right, meaning that they tend to be higher than the probabilities returned by SVM. We compare the raw probabilities in terms of a Mann-Whitney test and find a significant difference (p) for all the possible scenarios (panels A–D). (TIF) [file pone.0070153.s003.tif]
